# Insufficient Sleep Syndrome: Is it time to classify it as a major noncommunicable disease?

**DOI:** 10.5935/1984-0063.20180013

**Published:** 2018

**Authors:** Vijay Kumar Chattu, Sateesh M. Sakhamuri, Raman Kumar, David Warren Spence, Ahmed S. BaHammam, Seithikurippu R. Pandi-Perumal

**Affiliations:** 1 Faculty of Medical Sciences, The University of the West Indies, St. Augustine, Trinidad & Tobago.; 2 President, Academy of Family Physicians of India, New Delhi, India.; 3 Research Consultant, Dufferin Street, Toronto, ON M6K 2B4, Canada.; 4 University Sleep Disorders Center, College of Medicine and National Plan for Science and Technology, King Saud University, Riyadh, Saudi Arabia.; 5 Somnogen Canada Inc., College Street, Toronto, ON, M6H 1C5, Canada.

**Keywords:** Sleep Deprivation, Mortality, Morbidity, Epidemics, Public Health

## Abstract

Over the last three to four decades, it has been observed that the average total number of hours of sleep obtained per night by normal individuals have decreased. Concomitantly, global figures indicate that insufficient sleep is associated with serious adverse health and social outcomes. Moreover, insufficient sleep has been linked to seven of the fifteen leading causes of death. Additionally, current evidence suggests that sleep plays a significant role in determining cognitive performance and workplace productivity. There is a great need for a systematic analysis of the economic impact of insufficient sleep, particularly given current evidence that this phenomenon, as well as the poor sleep hygiene practices which produce it, is increasing worldwide. This paper takes the view that health authorities around the world need to raise the general awareness of benefits of sleep. There is considerable scope for research into both the public health impact as well as the macroeconomic consequences of insufficient sleep syndrome (ISS). Additionally, various models which estimate the undiagnosed burden of ISS on the GDP (gross domestic product) are needed to prioritize health issues and to highlight the national policies that are necessary to combat this medical problem. Sleep insufficiency has been declared to be a ‘public health epidemic’; therefore, we propose ISS as a potential noncommunicable disease. This review elaborates on this topic further, exploring the causes and consequences of insufficient sleep, and thus providing a perspective on the policies that are needed as well as the research that will be required to support and justify these policies.

## INTRODUCTION

Sleep is primarily a biological process that is as essential for survival as the need for food and drink. The American Academy of Sleep Medicine (AASM) and the National Sleep Foundation (NSF) recommends that school-age children should receive at least 10 hours of sleep daily while the minimum requirement for adults should be 7-8 hours^1^-^3^. The reality, however, is that large proportions of the population are sleeping far less than these recommended minimums. Population-based studies indicate that nearly 30% of American adults report sleeping an average of 6 or fewer hours per night, while 69% of high school students report having less than 8 hours of sleep on an average school night^4^-^7^.

During the last few decades, many studies have reported a strong association between sleep duration and mortality risk. These findings have shown that individuals sleeping between 7-9 hours at night experience the lowest risks for all-cause mortality when compared to those who sleep for shorter or longer periods^8^. In the United States (U.S.), insufficient sleep duration has been linked to 5 of the top 15 leading causes of death including cardio- and cerebrovascular diseases, accidents, diabetes mellitus, and hypertension^9^.

Even though the lack of sleep is thought of as an individual or a personal issue, insufficient sleep can have much wider adverse social and economic effects. Due to its potential negative impact on higher functions such as judgment, decision-making, and reaction time, sleep deprivation can increase the risk of fatal accidents and injury^10^,^11^. Historically some well-known catastrophes including the Chernobyl nuclear explosion, the Three Mile Island nuclear incident, the Exxon Valdez spill and the Space Shuttle Challenger tragedy have been linked with sleep deprivation^12^-^16^.

Insufficient sleep syndrome (ISS), also referred to as “chronic insufficient sleep,” “voluntary sleep curtailment,” “sleep reduction,” “sleep restriction,” “inadequate sleep,” or “sleep deprivation” was first recognized as a clinical syndrome in 1979 with its inclusion in the Diagnostic Classification of Sleep and Arousal Disorders (Association of Sleep Disorders Centers 1979)^17^.

The first important alert about the rising problem of insufficient sleep was reported in 1993 by the National Commission on Sleep Disorder Research: Wake up America, a National Sleep Alert^14^. The U.S. Congress mandated the commission in 1988. The Commission reported to the Secretary of the Department of Health and Human Resources^18^. The report indicated that millions are severely sleep deprived as a result of demanding work schedules and various other lifestyle factors. The adverse health effects of reduced sleep, as well as its secondary impact on job performance, traffic accidents, and reduced industrial productivity, has sent a wakeup call to find solutions for the problem of sleep deprivation and insufficient sleep^14^.

A recent study published by the Centers for Disease Control and Prevention (CDC)^19^ highlighted the issue of insufficient sleep; it was concluded that more than a third of American adults are not getting enough sleep on a regular basis. As a consequence of these findings, the CDC declared that insufficient sleep is a ‘public health epidemic’^19^.

Therefore, a new syndrome has recently been characterized called “insufficient sleep syndrome” (ISS), which is defined by hypersomnolence symptoms due to chronic sleep debt resulting from self-induced sleep restriction or wake extension^20^.

ISS affects all ages and both sexes. Nevertheless, it may occur more frequently among adolescents, in whom the strong developmental need for sleep is accompanied by social pressure and tendency to delay sleep, which often leads to chronic sleep restriction^7^. Moreover, cultural factors may influence sleep duration, with variations in sleep time of between six and eight hours per night being reported by students from different countries^21^. According to the recently released (2018) edition of the ICD-11 for Mortality and Morbidity Statistics (ICD-11 MMS), ISS was categorized as a distinct disorder under hypersomnolence disorders (7A26)^22^.

The American Academy of Sleep Medicine in the third edition of the International Classification of Sleep Disorders (ICSD-3) included ISS as a distinct disorder with clearly defined diagnostic criteria under “central hypersomnolence disorders”^20^,^21^. Diagnostic criteria include daily periods of an irrepressible need to sleep or daytime lapses into sleep for three months, with a duration of sleep shorter than expected for age, being present for at least three months^20^. Additionally, the criteria indicated clearly that the symptoms are not better explained by another untreated sleep disorder, the effects of medications or drugs, or themedical, neurologic, or mental disorder.

The WHO defines non-communicable diseases (NCDs), “as chronic diseases that tend to be of long duration and are the result of a combination of genetic, physiological, environmental and behaviors factors.”^23^ The WHO also adds “These diseases are driven by forces that include rapid unplanned urbanization, globalization of unhealthy lifestyles and population aging.”^23^.

ISS, which is recognized now as a distinct disorder by the ICSD-3, meets several criteria in the WHO definition of NCDs. It is chronic and related to environmental and behavioral factors. Genetic factors could also be involved, since recent data show that some people are more vulnerable to the consequences of sleep deprivation^20^,^24^-^27^. Additionally, ISS has a significant impact on health as discussed in this paper.

Considering the significant medical and mental consequences of ISS, it is prudent to raise a red flag to health planners about the high prevalence and significant detrimental effects of this new disorder. The importance of sleep and its disorders, in general, are under-recognized and under-estimated by health planners^21^. Therefore, the position taken in this paper is that sleep medicine specialists have a responsibility to bring this critical issue to light. Hence, we raise an important question for the healthcare community, should ISS be classified as a major NCD? In this paper, we review the effects of ISS on vital organs, the links between sleep loss and injury and death and in general will attempt to make the case that ISS is much more than a simple sleep disorder.

### Prevalence of insufficient sleep syndrome

Insufficient sleep is a global problem that is becoming increasingly common in today’s society. Compared to a few decades ago, significant changes in sleep culture have been observed worldwide. An argument could be made that at the top of the list of disruptive changes which separate the late 20^th^ and early 21^st^ centuries from almost all other centuries before them, have been the increasing adoption of 24/7 lifestyles, longer working hours and extended work shifts. This global trend has produced massive social and economic shifts, and additionally has had marked public health consequences, and foremost among these is the significant reduction in total sleeping hours that have occurred in both adults and children.

Studies from different parts of the world have shown an increased prevalence of insufficient sleep^28^,^29^. The cause of sleepiness in insufficient sleep syndrome is a voluntary restriction of daily sleep time to an extent that is less than the individual’s specific biological sleep requirements. The most common cause of excessive daytime sleepiness in modern society is chronic sleep deprivation. The prevalence of excessive daytime sleepiness has ranged widely in many studies worldwide (Australia, USA, New Zealand, Poland, Asia, Korea, Saudi Arabia, Japan), from 2.5% to 26%^30^-^46^.

Among the various surveys done in different countries, there was a significant proportion of participants reporting insufficient sleep. Insufficient sleep was reported by 23% of a representative survey from Japan^47^, 12% of a representative survey from Sweden^48^, and by 9% of a representative survey from Finland^49^. In 2008, the CDC examined data from over 400,000 subjects throughout the U.S. and found that 11.1% reported that they had had insufficient rest or sleep every day during the preceding 30 days. Females (12.4%) were more likely than males (9.9%), and non-Hispanic blacks (13.3%) were more likely than other racial/ethnic groups, to report insufficient rest or sleep^5^. A recent study conducted among Korean adults (19 years and older) found that the prevalence of excessive day sleepiness was 11.9%^50^,^51^. A recent cross sectional Japanese study which used a web-based questionnaire to ask about health-related quality of life issues, found that respondents aged 20 to 25 years and who were either students or full time employees (11% of the sample), reported that they suffered from insufficient sleep syndrome^52^.

In a survey regarding the sleep habits of a representative sample of residents of five high-income Organization for Economic Co-operation and Development (OECD) countries (Canada, Germany, Japan, UK, and the USA), the National Sleep Foundation (2013) found that insufficient sleep was a commonly reported problem, thus confirming that the issue is not confined exclusively to the U.S., but is also present in these high-income countries (^Table 1)3^. Insufficient sleep is a global problem that is not limited to a particular group of people, a nation, a gender or a specific age group. In fact, it is a new global health problem, and one that is being observed among millions of adults and children worldwide^53^.

**Table 1:** Proportions of the population sleeping less than seven hours.

	US	UK	Germany	Japan	Canada
Less than 6 hours	18%	16%	9%	16%	6%
6 to 7 hours	27%	19%	21%	40%	20%

**Source:** Sleep data based on data from the National Sleep Foundation (2013) representative survey.

It is also recognized that insufficient sleep is a significant problem among younger school-aged children, and that the problem tends to increase as children reach their high school years. In a 2015 study the CDC analyzed the cumulative results from Youth Risk Behavior Surveys (YRBSs). The surveys, which were carried out on students in large urban school districts, as well as on others at the national and state levels, sought to establish the prevalence of short sleep duration (<9 hours for children aged 6-12 years and <8 hours for teens aged 13-18 years) on weekdays among middle school and high school students in the US^54^. The investigation found that the prevalence of short sleep durations among middle school students was 57.8%, with state-level estimates ranging from 50.2% to 64.7%. On the other hand, a higher prevalence of short sleep duration was reported among high school students in the national YRBS (72.7%)^54^.

According to a National Sleep Foundation (2006) survey^6^, more than 87% of high school students in the U.S. reported getting far less than the recommended hours of sleep, and, further, that the amount of sleep that they did get was decreasing, thus posing a serious threat to their health and academic success. The problem of insufficient sleep among adolescents and children is not limited to the U.S. and developed countries only. Current data support the conclusion that adolescents worldwide are not acquiring sufficient sleep. Studies have shown that the worldwide prevalence of sleepiness among adolescents ranges from 25% to 84%^7^,^55^-^58^. Additionally, several studies have reported that adults, children, and adolescents from families with low income or of racial or ethnic minorities may be at an even higher risk of poor-quality and insufficient sleep^21^,^59^.

### Causes of insufficient sleep

The high prevalence of habitual short sleep and its association with morbidity and mortality warrant the identification of risk factors for short sleep and interventions to increase sleep duration in those with insufficient sleep. In 2006, the Institute of Medicine report emphasized that it is impossible to identify the relative contributions of pathological versus behavioral factors which lead to sleep loss^60^. In fact, it tends to be the rule and not the exception that sleep deprivation results from a combination of factors, and not any single factor^61^,^62^. For example, insufficient sleep is more common in individuals who do the night shift work or who work more than 40 hours per week. This tendency is slightly greater among females. Patients often report daytime sleepiness that interferes with their activities and functioning^61^,^62^. While work schedules are an important environmental contributor to reduced durations of sleep, the demands of work do not have the same effect on all individuals. As the research by Grandner below shows, various personal or psychological factors may modulate the impact that environmental stressors, such as atypical increased work demands, have on an individual.

Grandner has summarized a Social Ecological Model of sleep^63^. According to this model; some behaviors are genetically and intrapersonally driven. Others are socially driven, yet orchestrated by the environment. In doing so, they are subjected to interpersonal and societal factors^63^. Race or ethnicity may sometimes interact with this already complicated network of influences. Interactive effects have been found for instance between race and type of industry, and these effects in turn may limit sleep duration. Jackson et al. found for instance that Asians were more likely to report having short sleep durations when compared to Caucasians, but that this difference was greatest in the finance/information and healthcare industries^64^-^66^. In a study of Black and Caucasian racial disparities in sleep quality (analyzed by industry and occupation), it was found that the type of job an individual has produces differential effects on sleep duration and that these were mediated by the person’s race. The investigators found that as Blacks attained greater responsibility and higher paying jobs the duration of their sleep decreased, whereas their Caucasian counterparts showed the opposite pattern^66^.

Behavioral causes of sleep deprivation include a number of issues which may range from a person’s decision to restrict sleep time in pursuit of other activities or consumption of stimulants such as coffee and tea close to bedtime^20^,^63^. The disruption of sleep cycles is also seen very commonly among shift workers and frequent business travelers^22^,^67^. The growing levels of stress and unrealistic targets and time pressures at workplaces have an adverse impact on sleep^21^. Sleep deprivation is also becoming very common among school-age children and adolescents as their schedules and demands are preventing them from having a sufficient sleep, although it is recommended these groups should try to sleep more than adults^7^,^25^,^68^,^69^.

### Consequences of insufficient sleep syndrome

Insufficient sleep can lead to serious consequences for almost all bodily organs and systems. However, cognitive impairment, obesity, hypertension, and insulin resistance (diabetes) are the most pronounced^53^. Additionally, immune function is reduced, and increases in systemic inflammation inflammatory markers occur^53^, and several hormones become upregulated^53^,^70^. Moreover, several epidemiological studies have revealed that shorter durations of sleep are associated with increased mortality^71^-^73^. Table 2 shows a summary of the major adverse effects of insufficient sleep^74^-^92^.

**Table 2:** A summary of the main consequences of insufficient sleep.

Complications	Effects	References
Daytime sleepiness	- Inadvertently fall asleep during sedentary activities,such as meetings, reading, watching television or movies, or while driving and increased risk for motor vehicle accidents	Komada et al.^92^
Emotional disturbances	- Results in a more negative mood, with reduced optimism and sociability. Complaints of pain were also observed - Worsens mood states in healthy adolescents, with females having heightened vulnerability	Haack and Mullington^77^ Short and Louca^83^
Effects on functions of the brain	- Cognitive impairment, prefrontal cortex dysfunction, Novelty detection, a mechanism that involves the frontal lobes, gets negatively affected - Memory disorders	Gosselin et al.^75^ Saletin et al.^82^
Effects on the structure of the brain	- Reduction of cells in the dentate gyrus of the hippocampus - Structural changes in the cortical neurons- Degeneration of locus ceruleus neurons	Guzman-Marin et al.^76^ Roman et al.^81^ Zhang et al.^84^
Effects on body weight	- Weight gain during insufficient sleep reverses when normal sleep is resumed - Decrease of appetite-suppressing hormone leptin while levels of ghrelin, a hunger	Knutson and Van Cauter^78^ Nedeltcheva et al.^79^
Glucose metabolism	- Glucose tolerance test shows a pre-diabetic state in otherwise normal persons - Changes in insulin sensitivity and body weight - Increased insulin resistance in diabetes	Robertson et al.^80^ Knutson and Van Cauter^78^
Cardiovascular system	- Hypertension, arrhythmia, oxidative stress,endothelial dysfunction, inflammation, and metabolic disorder in coronary heart disease patients - Coronary heart disease	Aldabal & Bahammam^53^
Reproductive system	- Impairment of sperm health	Liu et al.^85^
Genes linked with immune and inflammatory processes	- Fraternal twins have shown that resiliency and vulnerability to sleep loss are highly heritable - Variant in the ABCC9 gene that explains approximately 5% of thevariation in sleep duration - Genetic polymorphisms related to orexin signaling, are important for predicting an individual's vulnerability to overeating and gaining weight when sleep deprived	Kuna et al.^86^ Allebrandt et al.^87^ Spaeth et al.^88^
Circardian rhythms	Reduction in circardian transcripts in whole blood	Archer & Oster^89^
Immune System, Inflammation and Infection	- Decrease antibody production following influenza vaccination - Dampened the normal circadian T-cell function and regulation - Associated with a 1.39 relative risk of developing pneumonia - Alterations in interleukin 6 and tumor necrosis factor alpha - Leading to cardiovascular disease, insulin resistance, and osteoporosis	Bollinger et al.^90^ Patel et al.^91^ Irwin et al.^74^

In general, the consequences of insufficient sleep syndrome are often under-recognized^93^. Some of them include adverse performance effects at school and in the labor market. According to Kochanek et al., insufficient sleep duration has been linked with seven of the fifteen leading causes of death in the U.S.^9^ Chronic sleep deprivation has also been linked to increased risk of automobile and industrial accidents, declining job performance, and decreased sociability^94^. Recently, insufficient sleep has been shown to alter gene expression in human blood cells and to reduce the amplitude of circadian rhythms in gene expression^95^.

**Figure 1:**
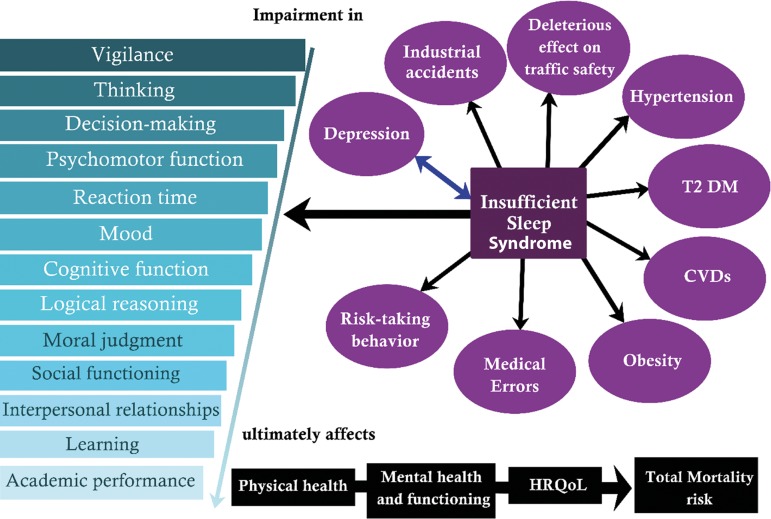
An accumulating amount of evidence has identified insufficient sleep as the first cause for a number of direct health consequences: These include impairments in physiological functioning, mental/cognitive deficits, and mood or emotional effects. Other downstream sequelae include problems in the workplace and personal lives of affected individuals.

Figure 1 shows a summary of the important adverse health outcome associated with insufficient sleep.

Existing evidence suggests that besides promoting health and well-being, normal sleep plays a vital role in determining cognitive performance and workplace productivity. Conversely, a lack of sleep increases the likelihood of traffic accidents, industrial accidents, medical errors and loss of work productivity^10^,^11^. While insufficient sleep has been shown to have a detrimental effect on all age cohorts, sleep deprivation among children and adolescents may trigger irreversible long-term consequences. There is now substantial evidence for an association between quality and quantity of sleep and school performance and cognitive ability among school-aged children and adolescents^96^-^98^.

### Economic Impacts

**Figure 2:**
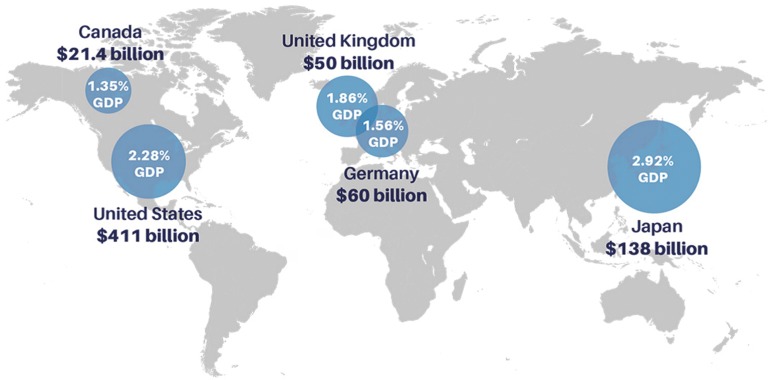
Economic costs of insufficient sleep across five OECD countries.

**Source:** RAND Europe Analysis.

The adverse effects of insufficient sleep are not limited to their effects at the individual level but also generalize to the community. Insufficient sleep among the population is associated with substantial economic losses, with adverse effects on economic output and labor productivity. It is estimated that up to $680 billion is lost each year across five OECD countries due to insufficient sleep^3^,^99^. While the exact contribution of various personal factors (work, social or family activities) to insufficient sleep, and their subsequent costs in terms of accidents and illnesses, are difficult to identify, the overall economic cost of sleep loss has been analyzed by the Deloitte consulting firm. The economic predictions by RAND Europe Analysis indicate that, the U.S., due to the size of its economy, sustains by far the highest annual economic loss (between $280 billion and $411 billion currently, depending on the scenario) followed by Japan (between $88 billion and $138 billion)^99^. However, relative to the scale of their overall economies, the estimated loss for Japan is larger than for the U.S. (between 1.56% to 2.28% for the U.S. and 1.86% to 2.92% for Japan, respectively). These financial loss estimations also appeared to be significant in the UK (1.36% to 1.86%), Germany (1.02% to 1.56%) and Canada (0.85% to 1.56%) as shown in Figure 2 ^99^.

### Proposed solutions and research agenda - call for action

The AASM, Sleep Research Society and the CDC initiated the National Healthy Sleep Awareness Project in 2013, aiming to improve public health by promoting adequate sleep^100^.

Considering the impact of sleep insufficiency on health, we propose including ISS as one of the major noncommunicable diseases. This proposal needs to be addressed seriously at the global level. National governments should be committed to deal with insufficient sleep by implementing various policies and enacting relevant laws to ensure proper sleep for teenagers and employees. National standards should be established in a number of areas. These should include the implementation of required daily start times for formiddle and high school students that are later in the day; stronger regulation of work hours and schedules should be implemented; the public should be educated regarding the impact of electronic media on sleep; and daylight saving time should be eliminated^101^.

According to the joint report of WHO and World Economic Forum, over the period 2011-2025, the cumulative lost output in low- and middle-income countries (LMICs) associated with the four NCD conditions (Diabetes, CVDs, Cancers and chronic respiratory diseases) that are the focus of the UN High-Level Meeting is projected to be more than US$ 7 trillion, which is an average of US$ 500 billion/year^102^. When compared to the economic burden due to the impact of insufficient sleep as described above, the financial loss to the countries due to direct and indirect causes of insufficient sleep is enormous. These considerations support the conclusion that insufficient sleep syndrome should be classified as an additional and legitimate entity among the traditionally recognized list of major NCD’s, particularly in view of its significant contribution to the economic loss impacting the GDPs of the countries. We propose that the public health importance of sleep needs to be emphasized as part of the NCD agenda at the national and, international and global levels.

## RECOMMENDATIONS

There is an acute shortage of systematic analysis of the economic impact of insufficient sleep, particularly given the evidence that rates of insufficient sleep are increasing worldwide. At the individual level, there is a great need to advocate publicly the importance of sleep to overall health, and that sleeping habits and daily routines should be adjusted accordingly. This advocacy should include specific recommendations for the establishment of a consistent wake-up time, the limiting of time spent in bed on activities other than sleeping (e.g., Watching TV, using mobile devices, or working), restricting the use of electronic devices before bedtime and avoiding consumption of substances that may impair sleep quality (e.g., caffeinated beverages, nicotine, and others). Additionally, it should be recommended that exercise be included as a daily habit, inasmuch as exercise is known to be associated with improved sleep outcomes^103^,^104^.

Concerning the working population, it is recommended that employers should promote the importance of sleep health to their employees; to help employees achieve better sleep outcomes by providing facilities and snooze-friendly policies; to discourage the extended use of electronic devices; to the variability of working hours and to maximize employees’ control over their schedules.

Public health authorities around the world need to raise the general awareness of benefits of sleep; to encourage employers to pay attention to sleep issues, to introduce delayed school starting times and, finally, to make use of existing workplace mandates and their enforcement. There needs to be a strong emphasis on public sleep health education and the inclusion of sleep as an essential component of healthy living through various public policies will protect against the morbidities and mortalities due to insufficient sleep.

There is an excellent scope for research in this area to estimate the macroeconomic effects of insufficient sleep and to use available models to determine the undiagnosed burden of insufficient sleep on the population in terms of GDP. Progress in this area will provide greater insights into how to improve awareness of the importance of sleep hygiene practices and how to incorporate this awareness into national policies.
